# The Variable CTCF Site from *Drosophila melanogaster* *Ubx* Gene is Redundant and Has no Insulator Activity

**DOI:** 10.1134/S1607672922040044

**Published:** 2022-08-29

**Authors:** A. N. Ibragimov, O. V. Bylino, O. V. Kyrchanova, Y. V. Shidlovskii, R. White, P. Schedl, P. G. Georgiev

**Affiliations:** 1 Institute of Gene Biology of the Russian Academy of Sciences, Moscow, Russia; 2 University of Cambridge, Cambridge, United Kingdom; 3 Princeton University, Princeton, USA

**Keywords:** insulators, CTCF, Bithorax complex, Ubx

## Abstract

CTCF is the most thoroughly studied chromatin architectural protein and it is found in both *Drosophila* and mammals. CTCF preferentially binds to promoters and insulators and is thought to facilitate formation of chromatin loops. In a subset of sites, CTCF binding depends on the epigenetic status of the surrounding chromatin. One such variable CTCF site (*vCTCF*) was found in the intron of the *Ubx* gene, in close proximity to the *BRE* and *abx* enhancers. CTCF binds to the variable site in tissues where *Ubx* gene is active, suggesting that the *vCTCF* site plays a role in facilitating contacts between the *Ubx* promoter and its enhancers. Using CRISPR/Cas9 and *attP/attB* site-specific integration methods, we investigated the functional role of *vCTCF* and showed that it is not required for normal *Drosophila* development. Furthermore, a 2161-bp fragment containing *vCTCF* does not function as an effective insulator when substituted for the *Fab-7* boundary in the Bithorax complex. Our results suggest that *vCTCF* function is redundant in the regulation of *Ubx*.

Parasegment-specific expression of the *Ubx*, *abd-A*, and *Abd-B* homeotic genes in the *Drosophila* *melanogaster* Bithorax complex (BX-C) is controlled by nine autonomous regulatory domains, which are separated by special elements called boundaries or insulators [1]. Boundaries ensure autonomy by blocking contacts between regulatory elements in one domain with regulatory elements in adjacent domains. Boundaries can also prevent enhancers from interacting with promoters [2–4]. In addition to insulator activity, some boundaries have an ability to specifically interact with their target gene in BX-C, enabling enhancers in distant regulatory domains to stimulate their target promoter [5]. These properties of the boundaries ensure correct parasegment-specific expression of the BX-C genes during *Drosophila* development. Consistent with this idea, *Fab-6, Fab-7,* and *Fab-8* were shown to specifically interact with the promoter upstream region of *Abd-B* gene [6]. It is likely that this interaction determines the correct topological positioning of the corresponding regulatory domains (*iab5 – iab7*) with *Abd-B* promoter in parasegments 10–12.

Most of the BX-C boundaries contain binding sites for *Drosophila* CTCF (dCTCF), and these sites are important for the insulator activity of these boundaries (Fig. 1) [7]. In the intron of the *Ubx* gene 30 kb downstream from the promoter, a variable dCTCF binding site (*vCTCF*) was identified (Fig. 1) [8]. dCTCF does not occupy this site in tissues where *Ubx* is inactive (imaginal discs of the first pair of legs), but binds to it when the *Ubx* gene is transcriptionally active (imaginal discs of the third pair of legs). Moreover, dCTCF binding to *vCTCF* is associated with changes in the topology of the *abx/bx* regulatory domain: in tissues where *Ubx* is active an increase in the frequency of *vCTCF* contacts with the *Ubx* promoter is observed [8]. A model was proposed according to which binding of dCTCF to *vCTCF* facilitates tissue-specific interaction of the *abx*, *BRE* enhancers with the *Ubx* promoter [9, 10]. The aim of this study was to test this hypothesis.

**Fig. 1.**
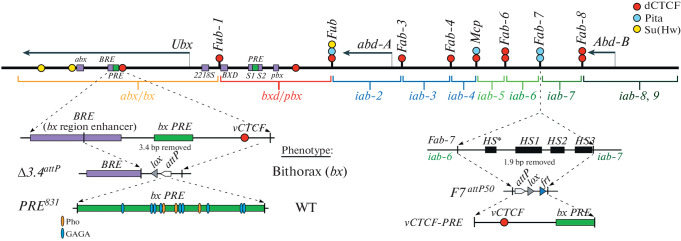
Schematic organization of the genes and regulatory domains in BX-C. *abx/bx, bxd/pbx* and *iab-2–iab-8* domains responsible for the regulation of *Ubx, abd-A,* and *Abd-B* genes and for the development of parasegments 5-13/T3-A8 segments are shown. *Ubx* embryonic enhancers are shown as purple boxes. The lines with colored circles mark boundaries. The binding sites for insulator proteins dCTCF, Pita, and Su(Hw) are shown as red, blue, and yellow circles. On the lower part of the figure, regulatory regions containing dCTCF variable site and *Fab-7* boundary, as well as their deletions are shown. *Fab-7* boundary Deoxyribonuclease I hypersensitive sites HS*, HS1, HS2, HS3 are shown as black rectangles on the coordinate bar. The bx PRE hypersensitive site is depicted as a green box, sites for Pho and GAGA-factor proteins as orange and blue ovals. *attP, lox,* and *frt* sites used for genetic manipulations are shown as white, gray, and blue triangles.

To study *vCTCF* function in enhancer-promoter interactions, we used the CRISPR/Cas9 system to delete a 3408-bp DNA fragment (3R:16701239..16704646) that spans the *vCTCF* site and the *bx* PRE (polycomb response element) 1 kb downstream, and in its place we introduced an *attP* site (Δ*3.4*^*attP*^*,* Fig. 1*).* Flies homozygous for Δ*3.4*^*attP*^ deletion show evidence of variable LOF transformations. The deletion transforms the anterior third thoracic segment toward the anterior second thoracic, a phenotype known as *bithorax* (*bx*) [11, 12]. In mutant flies the anterior third leg resembles the second leg, and in ~10% of flies anterior notal tissue is present on the dorsal surface of the third thoracic segment (Fig. 2). These transformations are caused by a disruption in the interactions of enhancers downstream of *vCTCF* with *Ubx* promoter. The Δ*3.4*^*attP*^ deletion overlaps with a previously described 9.5 kb deletion, *bx*^*34e-prv*^. Like Δ*3.4*^*att*^, it also has a variable *bx* phenotype which is caused by a decrease in *Ubx* expression in the imaginal discs of segment T3 [11]. Next, we used *attP* site in Δ*3.4*^*attP*^ as an integration platform to find minimal element that can rescue the mutant phenotype. We carried out *attP-attB* mediated integration of the 831-bp *bx* PRE fragment (*PRE*^*831*^, 3R:16702487..16703317) into Δ*3.4*^*attP*^ deletion and discovered that *PRE*^*831*^ completely reverts *bx* phenotype to wild type. This finding suggests that *vCTCF* is redundant, while *bx* PRE may play a role in facilitation of enhancer-promoter interaction.

**Fig. 2.**
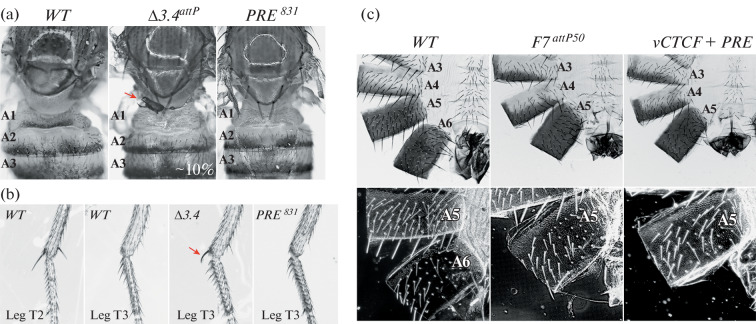
(a) Phenotypic comparison of T3-A1 tergites of *wt,* Δ*3.4*^*attP*^ and *PRE*^*831*^ flies. Δ*3.4*^*attP*^ has a variable phenotype, ~10% of flies have an enlarged A1 segment, a subset of dorsal T3 cells (marked with a red arrow) are transformed toward mesonotum, while their neighbors are untransformed. *PRE*^*831*^ integration restores the mutant phenotype to wild type. (b) Phenotypic comparison of T3 legs of *wt,* Δ*3.4*^*attP*^ and *PRE*^*831*^ flies. In wild type flies, T2 legs have a pair of long bristles, which are absent on T3 legs. Δ*3.4*^*attP*^ flies develop one long bristle on T3 legs (marked with a red arrow), which indicates a partial transformation toward T2. T2 legs of *PRE*^*831*^ flies look wild type. (c) Bright and dark field images of abdominal cuticle of *wt, Fab-7*^*attP50*^*, vCTCF+PRE* males. In wt males, A7 segment is absent, A6 sternite is banana-shaped and has no bristles, while A5 sternite is rectangular and covered with bristles. A5 tergite is completely covered with trichomes, while A6 has bristles only along anterior and ventral margins (see dark field). In *Fab-7*^*attP50*^ males, A6 segment is transformed toward A7 (does not develop) due to the fusion of *iab-6* and *iab-7* regulatory domains. *vCTCF+PRE* males also do not develop A6 segment.

In order to test *vCTCF* insulator activity we used *Fab-7*^*attP50*^ replacement platform (Fig. 1). In this platform, *Fab-7* boundary is removed, resulting in the fusion of *iab-6* and *iab-7* regulatory domains. This leads to ectopic activation of the *iab-7* regulatory domain in PS11, which in turn results in the loss of the sixth abdominal segment in adult males [13–15]. It was demonstrated previously that PREs are often located in close proximity to insulators and contribute to the formation of a functional boundary [16, 17]. Therefore, a fragment containing both *bx* PRE and *vCTCF* in reverse orientation, *vCTCF+PRE* (2161-bp, 3R:16702487..16704647) was tested in *Fab-7*^*attP50*^. We found that the 6^th^ abdominal segment is still missing in males carrying *vCTCF+PRE* insertion. This finding indicates that the *vCTCF+PRE* sequence does not have insulator activity.

Altogether, our data do not support a model in which *vCTCF* is a necessary mediator of enhancer-promoter interactions in *abx/bx* domain. Moreover, the data suggest that the *bx* PRE may play that role. However, further research is needed to explore the functions of this element in *Ubx* regulation. Since the loss of the *bx* PRE leads only to a variable LOF phenotype, it can be assumed that, in contrast to the *Abd-B* enhancers, *Ubx* enhancers are much more autonomous and less dependent on other regulatory elements to form appropriate promoter contacts.
