# Erratum to: **The Variable CTCF Site from** *Drosophila melanogaster* **Ubx Gene is Redundant and Has no Insulator Activity**

**DOI:** 10.1134/S1607672955340024

**Published:** 2023-02-14

**Authors:** A. N. Ibragimov, O. V. Bylino, O. V. Kyrchanova, Y. V. Shidlovskii, R. White, P. Schedl, P. G. Georgiev

**Affiliations:** 1 Institute of Gene Biology of the Russian Academy of Sciences, Moscow, Russia; 2 University of Cambridge, Cambridge, United Kingdom; 3 Princeton University, Princeton, USA

The article “The Variable CTCF Site from *Drosophila melanogaster* Ubx Gene is Redundant and Has no Insulator Activity,” written by A. N. Ibragimov, O. V. Bylino, O. V. Kyrchanova, Y. V. Shidlovskii, R. White, P. Schedl, and Academician P. G. Georgiev, was originally published electronically in Springer-Link on August 29, 2022 without Open Access. After publication in volume 505, issue 1, pages 173–175 the authors decided to make the article an Open Access publication. Therefore, the copyright of the article has been changed to © The Author(s) 2022 and the article is forthwith distributed under the terms of a Creative Commons Attribution 4.0 International License (http://creativecommons.org/licenses/by/4.0/, CC BY), which permits use, duplication, adaptation, distribution and reproduction of a work in any medium or format, as long as you cite the original author(s) and publication source, provide a link to the Creative Commons license, and indicate if changes were made.

